# A Novel Surgical Approach to Lipomatous Tumours of the Deltoid Region

**DOI:** 10.1155/2010/495834

**Published:** 2010-03-09

**Authors:** Emad Al Absi, Tamanna Karim, Nigel Colterjohn, Michelle Ghert

**Affiliations:** Department of Surgery, McMaster University and Hamilton Health Sciences, Juravinski Cancer Center, Hamilton, Ontario, Canada L8V 5C2

## Abstract

Resection of large lipomatous tumours in the subdeltoid region remains technically challenging due to the risk of injury to the axillary neurovascular bundle. We describe a novel deltoid release and reinsertion technique for resection of large lipomatous tumours of the sub-deltoid region and report the functional and oncologic outcomes of six patients who underwent this procedure. Three cases were diagnosed histologically as atypical lipoma and three cases were diagnosed as lipoma. There was one local recurrence in a case of an atypical lipoma. Rotator cuff function was comparable to that of the contralateral side in all cases and the average Constant Score adopted by the European Shoulder and Elbow Society was 84 (range 81 to 92) out of 100. We conclude that patients with large sub-deltoid lipomatous tumours who undergo resection through a previously undescribed deltoid release and reinsertion technique have excellent functional outcome with a low risk for recurrence.

## 1. Introduction

The upper extremity is affected by bone and soft tissue neoplasms one-third as often as the lower extremity [1]. When soft tissue tumours occur in the upper extremity, these tumours characteristically occur in the shoulder girdle. In the setting of a benign or low-grade tumour, surgical excision requires a balance between the resection margin and optimal functional recovery of the shoulder.

Lipomatous tumours of the soft tissues are the most common soft tissue tumours and these lesions can occur at every age and at almost any anatomical location [2, 3]. The shoulder, however, is a region where these tumours may present difficulty in adequate resection due to the proximity of the tumour to neurovascular structures and the functional loss associated with periscapular muscle resection.

The deltoid muscle has a multipennate origin from the lateral third of the clavicle, the acromion process and the spine of scapula, and is the largest muscle of the shoulder girdle. Its function is of paramount importance in abduction and flexion. The axillary nerve exits in the quadrangular space below the lower border of teres minor, where it passes around posterior and lateral to the humerus on the deep surface of the deltoid muscle. The nerve then splits into anterior and posterior trunks, both of which run intimately with the deep surface of the deltoid muscle. Tumours in the sub-deltoid region are therefore particularly difficult to resect due to the risk of injury to the axillary nerve [4]. Various techniques of deltoid mobilization have been described, and these techniques vary in the approach and extent of detachment of the deltoid muscle from its origin or insertion [5–7].

The aim of this study is to report the surgical and functional outcomes of our patient population with sub-deltoid low-grade lipomatous tumours who underwent marginal resection of their tumours though extensive deltoid mobilization with preservation of the axillary nerve branches.

## 2. Methods

A retrospective review of a prospectively maintained database was performed. Review of the database was approved by our institution's Research Ethics Board. We identified seven cases of large lipomatous tumours located in the subdeltoid region treated at our institution between March 1997 and July 2007. One patient was lost to follow-up, therefore a total of six patients were included in the follow-up section of the study. Patient demographics, indication for surgery, pathology and operative records were reviewed. Functional shoulder assessment was performed using the Constant scoring system adopted by the European Society for Shoulder and Elbow Surgery [8, 9]. The Constant Score is a subjective scoring system which includes parameters such as pain, return to function, activities of daily living, range of shoulder joint motion and strength of abduction. Outcome variables included major and minor complications, axillary nerve and rotator cuff function, recurrence rates and functional scores as determined by an independent examiner at the latest follow-up visit.

## 3. Operative Technique

With the patient under general anaesthesia and placed in a beach-chair position, we utilized a standard anterior deltopectoral approach to the shoulder. After the incision was made through skin and subcutaneous tissue, dissection was carried out through the delto-pectoral interval after isolation of the cephalic vein medially. A section of the anterior deltoid was sacrificed with the vein if necessary. Exposure was carried proximally to the clavicle and distally to the insertion of the deltoid.

**Figure 1:**
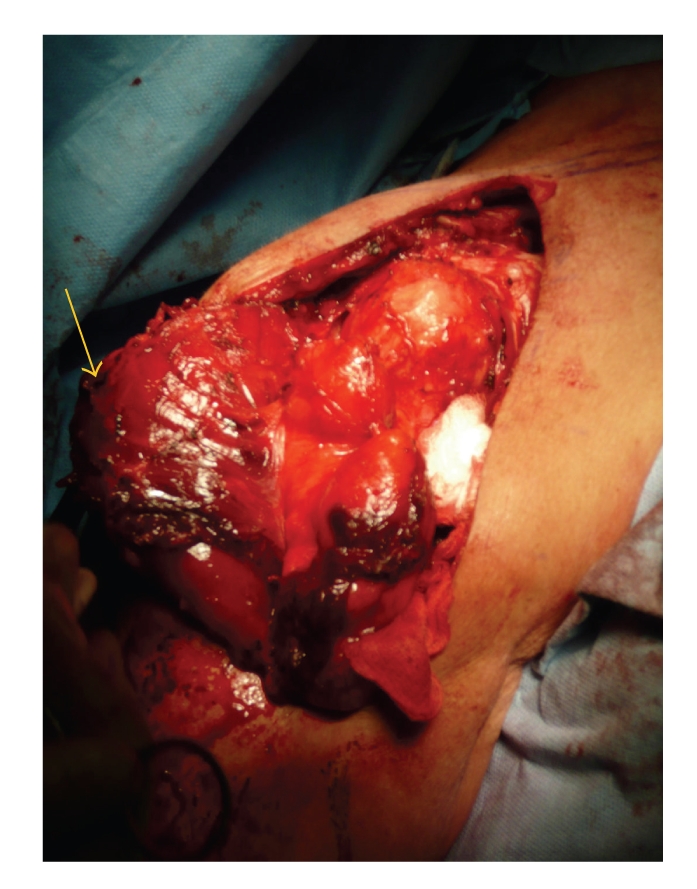
The deltoid muscle reflected inferiorly (arrow) and the lipomatous tumour exposed in the sub-deltoid space.

Mobilization of the deltoid was performed by releasing its multipennate origin with the use of a reciprocating saw from the anterior and lateral surface of the clavicle and laterally from the lateral edge of the acromion, and extending posteriorly to the quadrangular space as deemed necessary. The deltoid was also partially released from its insertion onto the humerus if necessary. The muscle was then reflected approximately 90 degrees (Figure 1) to allow for facility in dissection. The tumour was then dissected from its bed with care to preserve the axillary nerve and posterior circumflex artery, unless these structures were involved with tumour. All tumours were resected through the reactive zone of the tumour (FNLCC R1 resection, microscopic residual disease).

**Figure 2:**
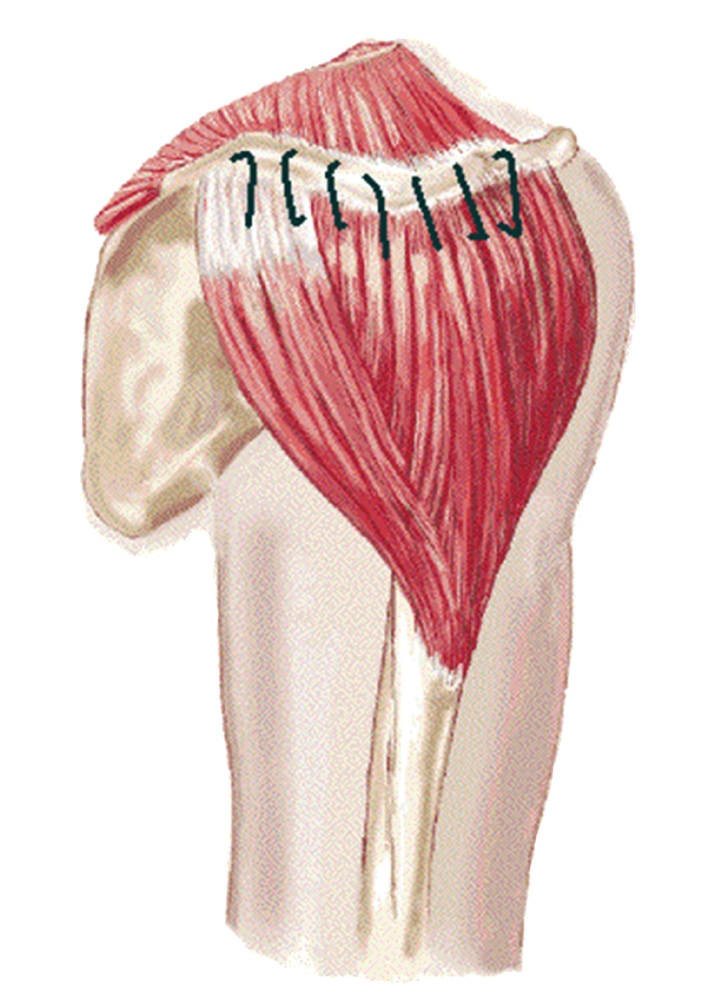
Reattachment of the deltoid muscle to its origin from the acromion and clavicle.

Closure was completed with reinsertion of the deltoid muscle to the acromion and lateral clavicle using multiple drill holes and attachment using nonabsorbable suture (Figure 2). The insertion site of the deltoid on the humerus was also repaired if necessary. The delto-pectoral fascia was approximated and closure of the subcutaneous tissue and skin was performed.

The operative arm was placed in a shoulder immobilizer for three weeks, with limited active abduction and flexion. Progressive active assisted and active exercises were then initiated and progressed gradually to deltoid and rotator cuff strengthening exercises.

## 4. Results

### 4.1. Demographics

**Figure 3:**
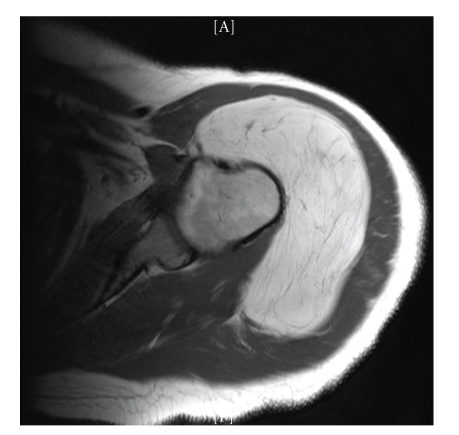
MRI Axial T1 Weighted image of the shoulder showing a large lipomatous tumour in the sub-deltoid region.

From 1997 to 2007, seven patients with sub deltoid lipomas or well-differentiated liposarcomas (M = 3, F = 4) underwent excision of the tumour (Table 1). One patient was lost to follow up. All patients presented with a painless slowly growing mass and one case had preoperative rotator cuff symptoms. The decision to operate was based on tumour growth as detected by serial clinical and radiological evaluations (Figure 3). Mean patient age was 68 years (range 48–87 years), and mean tumour size was 10.4 cm in maximum dimension (range 7–16 cm). The pathologic diagnosis was atypical lipoma in 4 cases and lipoma in 3 cases. Marginal complete resection (FNLCC R1) utilizing an extensive deltoid detachment with preservation of the axillary nerve branches was performed in five patients. One patient required partial axillary nerve resection for complete tumour excision. The mean duration of follow up was 51 months (range, 3–120 months).

### 4.2. Outcomes

There were no wound complications. Minimal atrophy of the anteromedial deltoid fibres was present in all six patients. One patient who required sacrifice of axillary nerve fibres had incomplete motor and sensory deficit of that nerve. A second patient had sensory dysfunction with persistent numbness and parasthesias in the distribution of the axillary nerve. 

The average Constant Score was 84 (range, 81–92), compared to 90 (range, 86–95) in the nonoperative shoulder (Table 2). All six patients regained full range of motion in shoulder flexion, lateral elevation and external rotation. One patient had full internal rotation to the interscapular region, while the remaining 5 patients had internal rotation to the twelfth thoracic vertebra. Rotator cuff function was comparable to the contra-lateral side in all patients. Three patients had mild weakness of the deltoid muscle and the remaining three patients had deltoid strength comparable to that of the contra lateral shoulder.

Local recurrence was documented in one case after nine years of the initial surgery. Final pathologic diagnosis in this case was atypical lipoma.

## 5. Discussion

Lipomatous tumours are the most common soft tissue neoplasms. These tumours can occur at every age and at almost any anatomical location in the body. The shoulder is one of the most common sites where these tumours may occur. Tumour excision at this site is challenging for the surgical oncologist in terms of proximity of the tumour to vital neurovascular structures and the subsequent need to balance the oncological resection and optional functional outcome.

The deltoid muscle has a multipennate origin from the clavicle, acromion, and the scapula. The axillary neurovascular bundle lies in close proximity to the muscle's under surface, rendering the approach to tumours of the sub-deltoid space at risk for neurovascular injury. The use of a conventional anterior or posterior approach to resect sub deltoid lipomatous tumours may not be adequate for complete resection of a tumour which extends beneath the muscle and adheres to the axillary nerve, or extends posteriorly to the quadrilateral space.

Martini [7] was the first group to describe a sub-deltoid approach to obtain wide surgical exposure of the sub-deltoid space. The authors described a V-shaped incision along the anterior and posterior borders of the deltoid where the distal tendon of the muscle was elevated off its insertion and elevated cranially with the overlying skin to expose the sub deltoid space, quadrilateral space, and the underlying neurovascular structures. The author's clinical series included 44 patients with osteomyelitis, neoplasms, recent fractures, and nonunions. Tamai et al. [5] reported the use of the same approach for treatment of two cases of soft tissue lesions with good results and normal axillary nerve function in both patients. However, despite the significant advantage of wide surgical exposure and protection of the axillary nerve, this approach unfavourably affects the deltoid muscle function postoperatively as it requires complete release of the tendon insertion distally.

All patients included in the current study had minor anteromedial deltoid atrophy. The deltoid atrophy is likely the result of minor denervation or interference with vascular supply of the muscle. However, the atrophy did not result in cosmetic or functional deficits. Two patients had axillary nerve dysfunction; one of them had a large tumour involving branches of the axillary nerve which were sacrificed in order to obtain adequate resection. Therefore, an unexpected axillary nerve injury occurred in 1 of 5 patients. These results are comparable to those of Tamai et al., who reported normal axillary nerve function in both patients reported [5].

Functional assessment unitizing the Shoulder Constant Score adopted by the European Society for Shoulder and Elbow Surgery showed that all six patients were free of pain and able to return to full activities. While the range of motion in forward flexion, lateral elevation, and external rotation was normal in all patients; five patients had limitation of internal rotation up to the T12 vertebra and one had symmetrical internal rotation to the interscapular at the T7 level. Minimal deltoid muscle weakness evidenced by difference in abduction strength compared to the contra-lateral side was present in half of the patients, but was not functionally significant as evidenced by the Constant Score.

In conclusion, we present a novel surgical approach to resection of lipomatous tumours of the sub-deltoid region. Our proximal-release approach results in excellent functional outcomes with a low risk of local recurrence and injury to the axillary nerve.

## Figures and Tables

**Table 1:** Patient and tumour characteristics.

Patient	Age	Gender	Size (max dimension, cm)	Length of follow-up (months)	Pathological diagnosis
1	79	Male	16	106	Lipoma
2	87	Female	7	120	Atypical lipoma
3	70	Male	8	20	Lipoma
4	48	Female	10	19	Lipoma
5	59	Female	—	—	Atypical lipoma
6	72	Male	15	37	Atypical lipoma
7	62	Female	9.5	3	Atypical lipoma

**Table 2:** Functional outcomes.

Patient	Constant		Range of motion		Abduction strength	
	score		External rotation	Internal rotation	(lbs)	
	Operative shoulder	Contralateral shoulder			Operative shoulder	Contralateral shoulder

1	84	89	Symmetrical	T12 vertebra	10–12	13–15
2	81	86	Symmetrical	T12 vertebra	7–9	10–12
3	92	95	Symmetrical	Interscapular (T7)	15–18	19–21
4	81	86	Symmetrical	T12 vertebra	7–9	13–15

5			Lost to follow-up			

6	83	95	Symmetrical	T12 vertebra	7–9	19–21
7	81	NA	Symmetrical	T12 vertebra	10–12	NA
